# A longitudinal study of infants’ early speech production and later letter identification

**DOI:** 10.1371/journal.pone.0204006

**Published:** 2018-10-10

**Authors:** Kelly Farquharson, Tiffany P. Hogan, Lesa Hoffman, Jun Wang, Kimber F. Green, Jordan R. Green

**Affiliations:** 1 School of Communication Science and Disorders, Florida State University, Tallahassee, Florida, United States of America; 2 Department of Communication Sciences and Disorders, MGH – Institute of Health Professions, Boston, Massachusetts, United States of America; 3 Child Language Doctoral Program, University of Kansas, Lawrence, Kansas, United States of America; 4 Department of Biomedical Engineering, University of Texas-Dallas, Richardson, Texas, United States of America; 5 Callier Center for Communication Disorders, University of Texas-Dallas, Dallas, Texas, United States of America; 6 Kimber Green Therapies, Boston, Massachusetts, United States of America; Hangzhou Normal University, CHINA

## Abstract

Letter identification is an early metric of reading ability that can be reliability tested before a child can decode words. We test the hypothesis that early speech production will be associated with children’s later letter identification. We examined longitudinal growth in early speech production in 9 typically developing children across eight occasions, every 3 months from 9 months to 30 months. At each occasion, participants and their caregivers engaged in a speech sample in a research lab. This speech sample was transcribed for a variety of vocalizations, which were then transformed to calculate consonant-vowel ratio. Consonant-vowel ratio is a measure of phonetic complexity in speech production. At the age of 72 months, children’s letter knowledge was measured. A multilevel model including fixed quadratic age change and a random intercept was estimated using letter identification as a predictor of the growth in early speech production from 9–30 months, measured by the outcome of consonant-vowel ratio. Results revealed that the relation between early speech production and letter identification differed over time. For each additional letter that a child identified, their consonant-vowel ratio at the age of 9 months increased. As such, these results confirmed our hypothesis: more robust early speech production is associated with more accurate letter identification.

## Introduction

Early language skills such as phonological awareness [1, 2, 3, 4], vocabulary [5, 6, 7, 8], syntax [9] and letter knowledge [10, 11, 12] are predictive of reading performance. Thus, children with weak early language skills are at risk for reading impairments. Unfortunately, these ‘early’ language skills are not measured reliably until 3–5 years of age [13]. In this study, we hypothesize that an earlier language ability, speech production, measured in infancy and toddlerhood will be associated with later letter knowledge. If confirmed, early speech-based metrics have the potential to identify children at risk for reading impairment well before other language skills can be reliably measured.

Early speech production is perhaps best defined as “utterances of speech as well as other (nonmeaningful) utterances that could be said to be phonotactically well-formed for some natural or potential natural language” [14]. Early speech production is an informative metric that is directly related to later outcomes, such as lexical development [15, 16, 17], phonological awareness [18], letter-sound correspondence [9] and later reading outcomes [19, 20, 21, 22]. In fact, early speech production is related to how young children learn new words [17] as well as the strength of their phonological memory [23]. However, the methods by which early speech production is measured in each of these studies is variable. For instance, some researchers have measured the proportion of vocalizations that include a consonant and have reported that this measure of early speech production is a robust predictor of vocabulary development [17, 23, 24, 25]. Scarborough (1990) reported that children’s early speech production accuracy—measured by percentage of consonants correct—predicted their knowledge of later letter-sound correspondences. Importantly, in her study, Scarborough (1990) found that children with many consonant errors in their early speech were more likely to be later diagnosed with a reading impairment. Similar reports have shown that speech sound errors related to a developmental sequence of acquisition (e.g., early, middle, or late 8 phonemes; [26, 27]) in preschool is related to performance on phonological awareness tasks [18]. Finally, a robust line of work has examined a variety of speech-related measures and their relations to later reading outcomes [19, 20, 21, 22]. For example, Smith et al. (2008) found that pause time during speech production of 3-year-olds was related to their 3^rd^ grade reading comprehension and nonword decoding. In a related study, Lambrecht-Smith (2009) reported that children diagnosed with a reading impairment in 2^nd^ grade or later had fewer polysyllabic words in their early speech production. Additionally, these children had a lower number of different words, which is a measure of lexical diversity.

Lexical diversity, or vocabulary complexity, has significant relations to both early speech production and later literacy skills. Children who produce more complex phonetic forms in their babble and early speech production are predicted to have a larger expressive vocabulary [16]. Specifically, the proportion of vocalizations that include a consonant is a robust predictor of vocabulary development. Additionally, Stoel-Gammon (1989) showed that depressed vocabularies were evident in children who exhibited limited phonetic repertoires during early speech production. Finally, Oller, Eilers, Neal, and Schwartz (1999) reported that a delayed onset of canonical babbling, or the use of repeated consonant and vowel sequences, was related to small vocabularies in children at 18-, 24-, and 30-months-old. As such, there is evidence to support the relation between the development of the phonological system, measured by early speech production, and later language development, measured by vocabulary output. Indeed, vocabulary is a robust predictor of literacy skills [5, 6, 7, 8]; Murphy et al. (2016) reported that children with lower levels of lexical quality in preschool had weaker reading comprehension, listening comprehension, and word reading skills in their first grade.

These examples of less sophisticated speech production paired with later reading impairments points to a broad phonologically based issue that may begin with early speech production. It is plausible that deficits in both speech production and reading indicate overall weak phonological representations. Phonological representations refer to the storage of word-level phonological information in long-term memory [28, 29, 30]. The quality of the representations will determine the child’s speech production accuracy [30, 31, 32], vocabulary size [15], and word reading abilities [28, 33, 34, 35]. Links between early speech and reading skill may be mediated by emerging phonologic skills—both speech production and word reading rely on well-defined phonological representations. Although accurate word reading skills are not expected until a child receives formal schooling, there is mounting evidence that foundational skills necessary for literacy are built in infancy.

Thus, it follows that the strength of phonological representations is critical for mapping phonemes to orthographic, or letter, representations [4, 36, 37]. For example, Justice, Pence, Bowles, and Wiggins (2006) showed that children are more likely to know letters that corresponded with earlier acquired speech sounds (e.g., B and /b/) as compared to letters that corresponded with later acquired speech sounds (e.g., R and /r/). Similarly, Treiman, Weatherston, and Berch (1994) and McBride-Chang (1999) report that the name of the letter itself influences a child’s ability to learn it. That is, letter names that are comprised of a consonant-vowel sequence (e.g., /bi/ for B) are easier to learn than those comprised of a vowel-consonant sequence (e.g., /ɛf/ for F). These findings underscore the importance of phonological properties that influence letter name knowledge and how children decode and spell words [38, 39]. Two additional examples of this are spelling the word “wife” with the letter “Y” [40] or more easily decoding words that start with the letter B followed by the vowel /i/ because of the influence of the letter’s name (i.e., /bi/ as in “beach”; [41]).

Theoretical support for the relation between phonology and orthography is further outlined in the self-teaching hypothesis [42]. “The self-teaching hypothesis proposes that only the ability to translate a printed letter string into its spoken form offers a reliable means of independently identifying new letter strings” [43]. The hypothesis emphasizes the reciprocal relations between the development of phonological and orthographic representations [44, 45]. For instance, the phoneme /f/ can be represented orthographically with the letter “F” (as in *f**an*), or the letter sequences “PH” (as in *ph**one*) and “GH” (as in *lau**gh*). On the other hand, the letter “S” can be represented phonologically by the phoneme /s/ (as in *s**un*), the phoneme “sh” (as in *s**ure*), or the phoneme /z/ (as in *tree**s*).

According to the self-teaching hypothesis, children who have more frequent exposure to their respective phonology, via early speech production, and to letter patterns experience a “self-teaching” mechanism that leads to more successful reading experiences. During this process, children are strengthening mappings between phonological and orthographic representations. Thus, learning to correctly articulate speech sounds may augment the phonological knowledge necessary for learning letter names and sounds. Pursuant to the aims of the present investigation, we explored how the frequency and complexity of early speech production influences letter knowledge. If we confirm that there is a relation between these two constructs, this may provide additional support to the self-teaching hypothesis. Specifically, a relation between the development of early speech production and later letter knowledge may substantiate the reciprocal relation between phonological and orthographic knowledge, as outlined in the self-teaching hypothesis. Given the robust predictive relation between orthographic, or letter, knowledge and later word reading abilities [4,12,40, 41, 46, 47, 48, 49, 50, 51, 52, 53], it is prudent to explore avenues that may lead to earlier identification of literacy strengths and weaknesses.

In this study, we measured consonant growth in early speech production in typically developing infants longitudinally from 9–30 months of age, and then determined how that change was associated with the number of letters known at 72-months of age. Sounds present in a child’s early speech production comprise their phonetic complexity, or the phonological variation in babble [54, 55, 56]. Specifically, we asked, *what is the strength of association between the number of consonants produced during early speech production from 9–30 months and the ability to identify letters at 72 months (6-years-old)*? We hypothesized that the number of letters identified at 72 months would be associated with growth during early speech production from 9–30 months. Specifically, we predict that children who know more letters at 72-months-old will have produced more consonants early in their speech production trajectory, compared to children who know fewer letters at 72-months old. This study represents a first step in a line of inquiry aimed at determining early and sensitive measures for early identification of reading risk.

## Materials and methods

### Participants

This research was approved by the Institutional Review Board (IRB) at the University of Nebraska-Lincoln. Written informed consent was obtained from the parents of all participants. Participants were selected from a larger study examining motor development for speech production (see [57]). As a part of the larger study, families with infants were recruited to participate in data collection sessions every three months from 3-months-old to 30-months-old. Participants were recruited for the larger study through flyers posted in pediatrician’s offices and through ads placed in local newspapers. As an extension of the original study, participants were invited to continue data collection sessions every six months after 30 months of age. For the present study, we have included 8 occasions from infants from the ages of 9- to 30-months-old and one additional data collection session at 72-months-old.

The age range of 9- to 30-months was chosen for three primary reasons. First, we chose 9-months as our starting point to align with the time when most babies are in the canonical and/ or variegated babbling stages [58, 59, 60]. Second, this age range captured a common period of substantial growth in both phonological and lexical knowledge [61]. Third, and more practically speaking, our final occasion of 30-months corresponded with the final data collection session of the larger study. All data collection took place in a research lab.

Sample size was determined based on the number of children who continued participation until 72 months of age. The present study, then, reflects data from 9 typically developing infants (8 girls, 1 boy) for a total of 66 data points from ages 9 to 30 months. Of the 9 children, 6 had all 8 occasions, 1 had 7 occasions, 1 had 6 occasions, and 1 had 5 occasions. All infants were from English speaking families in the Midwest and were born at term with no neurological, vision, hearing, or physical impairments. Hearing was screened at every data collection session using an otoacoustic emissions procedure at 2, 3, 4, 5 kHz [62]. Occasionally, one of the infants did not pass the hearing screening because he/she was fussy, vocalizing, or congested, which are situations in which reliable otoacoustic emission readings cannot be obtained; however, no infants failed consecutive tests.

### Procedure

A trained and certified speech-language pathologist conducted all developmental testing, and collected all speech samples in a research lab (testing methods described below). Early speech samples were audio and video recorded and were obtained while the children were placed in a car seat and secured using a five-point harness. The car seat was attached to a dental chair and the child was positioned to face the primary caregiver. The infant’s primary caregiver, who was typically the mother, sat in front of the child.

Two communicative context conditions were examined during each 45-minute data collection session. The first condition was a natural “free-play” condition. Parents were provided with a basket of toys and instructed to play with their child. The second condition, which started approximately 10 minutes after free-play, was more structured than the first. The caregiver was given a set of toys and asked to take a few turns with the toy, and then to pause and wait to see what the child would do (“play” condition). Three different sets of toys were provided for each parent–child dyad. One set of toys was designed to elicit requesting, including toys in transparent containers. The second set of toys was designed to elicit joint attention and included picture books and “surprise” bags (bags with various toys inside). The third set of toys encouraged social interaction. Toys included pretend food, dolls, and pretend tools. Each set of toys was used for approximately five minutes before being replaced with the next.

#### Data transcription

The interactions between the parents and the child provided a broad sample of utterances that were transcribed for babble, vocalizations, words, and phrases. Audio and video recordings of the speech samples were transcribed by a trained speech language pathologist. The transcription method was developed based upon the work of Oller and Eilers (1989), but additionally motivated by Vihman and McCune (1994). Based upon this previously established coding scheme, utterances were coded as: word, possible word, single babble, reduplicated babble, or variegated babble [14]. A word production referred to words that were produced in context (e.g., “its Mickey”; “two”; “please”). Words had no more than one or two phonetic variations due to age-expected speech error patterns (e.g., substituting a /t/ for a /k/ phoneme, as in “tan” for “can”). Possible word referred to a production that had an identifiable referent (i.e., object in the room) or was accompanied by a gesture or imitation (e.g., “bir” for “bird”; “ru” for “run”). For these utterances, the transcriber was at least 50% confident that the child was attempting a word and at least one phoneme from the target word was required to be present. Productions were coded as a single babble if produced in a CV, VC, CVC, CCV, CCVC, VCC, CVV, or VCV pattern (e.g., “ooofff”; “dada”; “teebee”). Reduplicated babble contained the same C and V (i.e., gagagaga) and variegated babble contained different C and/ or V (i.e., gagu; gaba). For each data collection session included in the present study, at least 50 vocalizations are included; no utterances were excluded. A second researcher coded 20% of the vocalization samples across all ages. Interrater reliability was 87.5%

### Measures

To index each child’s level of speech development, we computed the consonant-vowel (CV) ratio (i.e., the number of consonants produced divided by the number of vowels produced) for each transcribed speech sample. CV ratios change as children add consonants and consonant clusters to their phonetic inventories, which are initially predominated by vowels. CV ratios have been demonstrated to increase with age and to adequately measure phonetic complexity [25, 63, 64, 65]. Further, CV ratios allow for a robust examination of speech production throughout development, particularly as the linguistic context changes. That is, as children move from a more primitive reduplicated babble into more advanced word production, this single measure captures the complexity of that linguistic and phonetic growth.

Our time-invariant predictor variable was letter identification. At age 72 months (an age at which most children should be able to identify all letters; [66], children were given the Woodcock Reading Mastery Test-Revised (WRMT-R; [67]) Letter Identification subtest. The letter identification subtest contains 51 upper and lower case alphabet letters in various fonts. Font variety allows for examination of both letter knowledge and general print exposure and adds a level of complexity to a letter naming task. Each child was presented with 3–6 items on multiple easel pages and asked to name the same letters in the same order. If the child responded with the correct name of the letter, the item is scored as correct. The split-half reliability for this subtest was *r* = 0.94 [67].

Our analytical approach used CV ratio as the outcome variable and letter identification as the predictor variable. In a sense, this approach can be considered “reverse prediction” in that, we are examining the extent to which individual differences in a later developing skill (i.e., letter identification) may be foreshadowed by differences in earlier developing skills. Practically speaking, this approach was necessary to examine the relation between our constructs of interest. We expand on the details of this approach below.

## Results

Descriptive statistics for early speech production (as measured by CV ratio) at ages 9 to 30 months and letter identification (letter ID) at age 72 months are presented in Table 1.

**Table 1:** Descriptive statistics for consonant-vowel ratio and letter identification.

Variable	N	Mean	Std Dev	Minimum	Maximum
CVRATIO9MOS	7	104.07	22.39	75.29	139.29
CVRATIO12MO	8	88.34	11.34	75.00	104.17
CVRATIO15MO	7	100.68	13.66	85.87	120.83
CVRATIO18	8	121.68	20.49	87.59	145.73
CVRATIO21	9	123.57	18.06	91.98	155.73
CVRATIO24	9	122.76	16.64	97.70	149.57
CVRATIO27	9	119.15	9.44	107.50	135.45
CVRATIO30	9	122.75	15.71	93.31	143.13
WRMT72	9	35.22	3.03	30.00	40.00

As reported in Table 1, the early speech production outcome of CV ratio was multiplied by 100 to offer more precision in reporting and to create a more interpretable scale (see [68] for a similar approach). To examine its longitudinal change across eight occasions from 9 to 30 months, we estimated multilevel models using residual maximum likelihood (REML) within Statistical Analysis Software (SAS) PROC MIXED. Although equivalent to traditional least squares estimation for complete data, our use of full-information REML estimation allows the inclusion of participants with missing outcomes under an assumption of missing at random (i.e., conditionally random given the participant’s other data). Further, REML provides unbiased random effects variances in small samples, and thus is preferable to maximum likelihood estimation for the present sample [69]. Accordingly, the significance of random effects was evaluated through −2LL differences between nested models (i.e., likelihood ratio tests), whereas the significance of fixed effects was evaluated via univariate and multivariate Wald tests using Kenward–Roger denominator degrees of freedom (which is also preferred for small samples).

**Fig 1:**
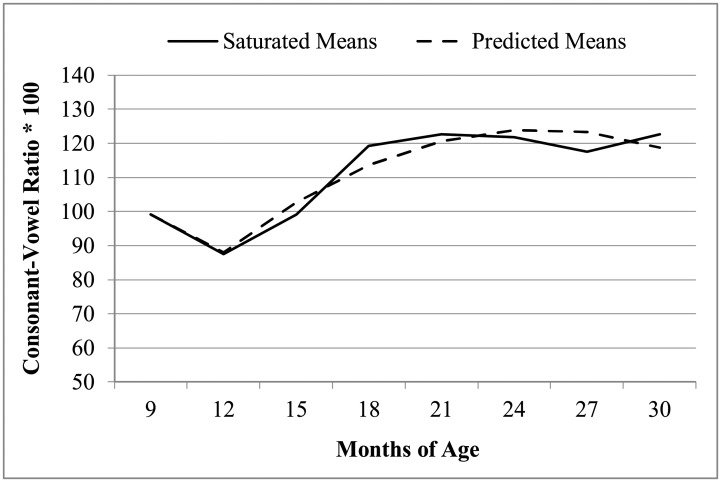
Predicted and observed means for early speech production scores across months- fixed quadratic, random intercept model.

To begin, an empty means, random intercept only model was estimated to partition the variance in CV ratio. The intraclass correlation (ICC) for the ratio of random intercept variance to total variance was .17, which indicates that 17% of the variance is from individual mean differences in children’s early speech production complexity over time. We also estimated a saturated means, random intercept model (with a separate mean for each occasion) to examine the shape of the average trajectory (Fig 1). To approximate this trajectory, fixed effects of linear and quadratic age (centered such that 0 = age 12 months) were then included. There remained a significant difference between the model-predicted trajectory and that given by the saturated means, *F*(5, 51) = 3.47, *p* < .01, which was largely due to a higher-than-predicted CV ratio at age 9 months. To capture this deflection, we added a piecewise linear slope (coded −3 for 9 months and 0 otherwise) to indicate the difference in change per month from ages 9 to 12 months. After doing so, the model-predicted trajectory did not differ significantly from the saturated means as desired, *F*(4, 51) = 1.03, *p* = .40. We then tested for individual differences in change and heterogeneity of variance over age, but none were found. More specifically, adding random linear or quadratic effects of age (and their covariances with the random intercept) did not significantly improve model fit, nor did adding heterogeneity of residual variance across age.

Thus, as shown in Fig 1, the best-fitting unconditional model for time included fixed effects of linear and quadratic age, a fixed slope deflection from 9 to 12 months, and a random intercept variance. As reported in the first set of columns in Table 2, at age 12 months (the intercept), the predicted mean consonant-vowel ratio was 89.63 with an instantaneous linear rate of growth of 5.55 per month that became less positive by twice the quadratic rate of change of −0.22 per month. In other words, the rate of growth in early speech production slowed down over time. The fixed 9-month slope deflection indicated that, relative to after 12 months of age, from ages 9 to 12 months the linear rate of growth was more negative by 11.14 per month. Overall, the effects related to change over age accounted for 42% of the residual variance.

**Table 2:** Results for longitudinal models predicting consonant-vowel ratio.

Model Parameters	Unconditional Model			Conditional Model		
	Estimate	SE	p <	Estimate	SE	p <
Fixed Intercept	89.63	5.20	.001	89.09	5.15	.001
Fixed Linear Age	5.55	1.08	.001	5.69	1.05	.001
Fixed Quadratic Age	-0.22	0.06	.001	-0.22	0.06	.000
Fixed 9-Month Slope Deflection	-11.14	3.06	.001	-11.29	2.95	.000
Letter ID by Intercept				1.64	1.35	.251
Letter ID by Linear Age				-0.55	0.22	.015
Letter ID by Quadratic Age				0.03	0.01	.021
Random Intercept Variance	75.03	50.43	.068	81.68	56.70	.075
Residual Variance	190.67	36.63	.001	176.99	34.67	.001

Note: SE = standard error, ID = identification.

**Fig 2:**
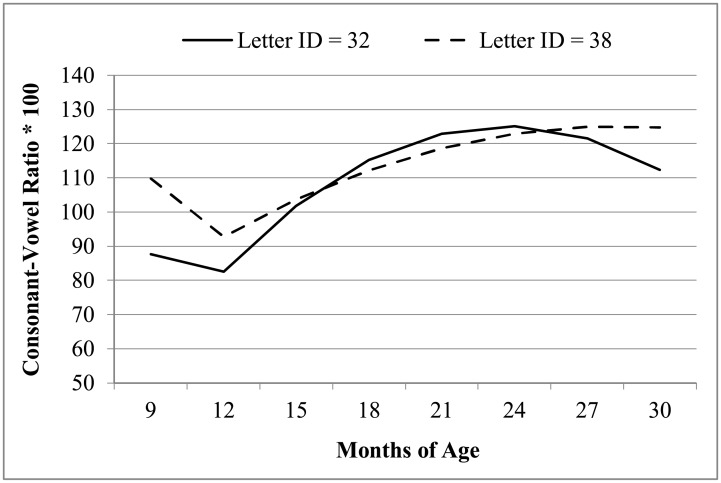
Predicted means of early speech production scores for children with high and low letter identification.

We then examined the effects of letter identification at 72 months (centered such that 0 = 35) in predicting early speech production over time—as a moderator of each fixed effect in the trajectory of CV ratio over age. The interaction of letter identification with the 9-month slope deflection was nonsignificant, and was thus removed. The final model is reported in the second set of columns in Table 2, in which the fixed effects for the intercept, linear age, and quadratic age refer to a reference child with a letter identification score of 35. For every additional letter identified, the quadratic rate of change was expected to be significantly less negative by 0.03. This pattern of interaction is depicted in Fig 2—children with better letter identification at age 72 months had less curvature in their earlier pattern of growth. Said differently, the effect of letter identification was largest in the earliest ages and diminished in an accelerated fashion over time. The interactions of letter identification by linear and quadratic age accounted for 7% of the remaining residual variance.

## Discussion

This study tested the hypothesis that early speech production is associated with later letter knowledge. To address this question, we examined the relation between growth in early speech production, indexed by consonant-vowel ratio, from 9 months to 30 months and letter identification skills at 72 months in 9 typically developing children. In support of our hypothesis, we observed that the trajectory of early speech production was related to later letter identification, and that early speech production was different for children with high levels of letter identification than for children with low levels of letter identification.

The association between early speech production and later letter identification was expected based on known links between phonologic and orthographic representations [36, 37]. When the connection between phonologic representations and orthographic representations is strong, children develop the appropriate letter-sound correspondences to become skilled readers [28, 31, 33, 35]. In the current study, children with higher letter identification also had more robust early speech production skills at 9-months-old, suggesting that an early, strong foundation in phonological abilities, via early speech production, is related to early, well-established orthographic representations.

Orthographic learning offers another explanation for the relations between early speech production skills and later letter identification abilities. The relation between phonology and orthography is reciprocal; just as children need to learn how phonology maps onto orthography, they also must to learn the different ways that orthography maps onto phonology. During early reading development, children are able to acquire rudimentary mappings from a word's letter sequence to its pronunciation and vice versa. Our data support a strong association between early speech production and later letter identification skills. Specifically, we found that early speech production—measured as consonant-vowel ratio—was related to letter knowledge such that greater early speech production at 9-months-old was related to knowing more letters later. This aligns with previous research that indicates that the onset of consonant production is related to later language outcomes, such as referential vocabulary [16]. This is particularly important as McGillon et al. (2017) indicated that this early measure of speech production indicates phonological readiness. Thus, it is plausible that children who use more consonants and consonant clusters during early speech production have had more exposure to and experience with those phonemes. These early speech production experiences lend themselves to a stronger foundation for learning orthographic information. As such, it is likely that early speech production skills may also serve as a sensitive early indicator for possible deficits in acquiring letter-sound correspondence skills (see also [9]).

In the current study, children with lower levels of letter identification ability exhibited less robust early speech production than did children with higher levels of letter identification. However, when examining early speech production growth patterns, children who knew fewer letters actually grew more over time. Although this may seem counterintuitive, it is reflective of the gap that exists between children with high and low letter knowledge. That is, children with higher letter knowledge started out with stronger CV ratios at 9-months-old when compared to children who knew fewer letters. As a result, children who knew fewer letters *needed* growth in early speech production in order to “catch up” to children who knew more letters. This result also highlighted the group differences at the beginning and ending of the observation period, at 9 and at 30 months. These end effects raise the possibility that the achievement gap between these groups may widen [70]. The apparent overlap in speech production skills between 15 and 24 months may be due to the wide variability in speech performance that is characteristic of children within this age range [55]. It is, therefore, plausible that differences are more visible on the “tails” of that unstable developmental time period. In addition, this gap is often seen with respect to early vocabulary [71, 72], phonological awareness, [3, 73] and reading development [72] and is often referred to as the Matthew Effect. In the Matthew Effect, “the rich get richer and the poor get poorer”. Although the children in this study were typically developing, we do see a gap in their letter knowledge that is also present in their early speech production as young as 9-months-old. This gap has clinical implications for current practices used for determining eligibility for early intervening services.

### Limitations

Although this study is an important step in determining earlier behavioral predictors of later reading outcomes, it has a few limitations. First, all children in the sample were typically developing, and therefore the range of variability in letter identification skills was restricted. Future studies will benefit from samples of children with less letter knowledge who have a higher risk of developing a reading impairment. Next, we used a single measure to refer to early speech production. Future work should consider the addition of multiple measures of speech production to create robust latent variables. In addition, more empirical data that support methods to predict later literacy skills from early speech production skills will have robust implications for clinical and theoretical frameworks. Finally, this study included a small sample of children. Although we were able to estimate robust statistical models due to having multiple time points per participant, we hope to conduct future research including larger samples of children. Future work should also consider examining both letter-name and letter-sound knowledge over time to determine the differential influences of early speech production on these two skills.

#### Summary

The results of our study support a connection between growth in early speech production abilities and later letter identification skills, which is a strong predictor of later word reading skills [10, 11]. Our study provides the necessary first step to establishing the validity of measuring early speech production to predict later reading achievement. Although additional work is needed to explore these relations in children with varying skill levels, there are robust clinical implications from this work. First, children who are late to develop consonants in their early babble may be at risk for a slower acquisition of later language skills, including vocabulary and letter knowledge. Second, speech-language pathologists (SLPs) are on the front lines of early identification of children who may be at risk for reading difficulties. Frequently, SLPs are the first professionals to work with a child who has had delayed development of speech and language. As such, the role of the SLP in the prevention, identification, and treatment of children at risk is paramount. In particular, our data point towards a potential need to measure speech and language skills at multiple time points for children in the birth-to-three range, and to consider measures that include spontaneous speech samples to augment the results of static standardized assessments. Finally, parents and other professionals who work with young children have an opportunity to build upon the speech and language skills of infants and toddlers by modeling strong speech production, expanding children’s utterances using grammatically correct language, and engaging in print-rich literacy activities, such as shared book reading.
